# Regulation of Cysteinyl Leukotriene Receptor 2 Expression—A Potential Anti-Tumor Mechanism

**DOI:** 10.1371/journal.pone.0029060

**Published:** 2011-12-15

**Authors:** Cecilia Magnusson, Astrid M. Bengtsson, Minghui Liu, Jian Liu, Yvonne Ceder, Roy Ehrnström, Anita Sjölander

**Affiliations:** 1 Cell and Experimental Pathology, Department of Laboratory Medicine, Lund University, Skånes University Hospital, Malmö, Sweden; 2 Pathology, Department of Laboratory Medicine, Lund University, Skånes University Hospital, Malmö, Sweden; 3 Clinical Chemistry, Department of Laboratory Medicine, Lund University, Skånes University Hospital, Malmö, Sweden; Fundação Oswaldo Cruz, Brazil

## Abstract

**Background:**

The cysteinyl leukotrienes receptors (CysLTRs) are implicated in many different pathological conditions, such as inflammation and cancer. We have previously shown that colon cancer patients with high CysLT_1_R and low CysLT_2_R expression demonstrate poor prognosis. Therefore, we wanted to investigate ways for the transcriptional regulation of *CysLT_2_R*, which still remains to be poorly understood.

**Methodology/Principal Findings:**

We investigated the potential role of the anti-tumorigenic interferon α (IFN-α) and the mitogenic epidermal growth factor (EGF) on *CysLT_2_R* regulation using non-transformed intestinal epithelial cell lines and colon cancer cells to elucidate the effects on the CysLT_2_R expression and regulation. This was done using Western blot, qPCR, luciferase reporter assay and a colon cancer patient array. We found a binding site for the transcription factor IRF-7 in the putative promoter region of *CysLT_2_R*. This site was involved in the IFN-α induced activity of the *CysLT_2_R* luciferase reporter assay. In addition, IFN-α induced the activity of the differentiation marker alkaline phosphatase along with the expression of mucin-2, which protects the epithelial layer from damage. Interestingly, EGF suppressed both the expression and promoter activity of the *CysLT_2_R*. E-boxes present in the *CysLT_2_R* putative promoter region were involved in the suppressing effect. CysLT_2_R signaling was able to suppress cell migration that was induced by EGF signaling.

**Conclusions/Significance:**

The patient array showed that aggressive tumors generally expressed less IFN-α receptor and more EGFR. Interestingly, there was a negative correlation between CysLT_2_R and EGFR expression. Our data strengthens the idea that there is a protective role against tumor progression for CysLT_2_R and that it highlights new possibilities to regulate the *CysLT_2_R*.

## Introduction

Prolonged inflammation is known to increase the risk of developing cancer [1], [2]. It has been estimated that 15% of all cancers can be accredited to infectious agents [3]. Tumor microenvironment has often been associated with infiltrating leukocytes in the tumor tissue and the surrounding stroma [4]. Moreover, patients with ulcerative colitis display a 30 to 50% increased risk of developing colon cancer [5]. Elevated levels of inflammatory mediators, such as the cysteinyl leukotrienes (cysLTs), have been found in these patients [6]. CysLTs is the collective name for LTC_4_, LTD_4_ and LTE_4_. They are lipid mediators derived from arachidonic acid through the 5-lipooxygenase (5-LO) pathway [7]. In addition to inflammatory bowel disease (IBD) [7], they have been implicated in the pathogenesis of several chronic inflammatory diseases, such as asthma, pulmonary fibrosis, and atherosclerosis [8], [9]. The known biological effects of cysLTs are mediated through two different G-protein coupled receptors (GPCRs): CysLT_1_R [10], which is the high affinity receptor for LTD_4_ and CysLT_2_R [11], which is the high affinity receptor for LTC_4_. Recently, an orphan GPCR denoted as GPR17 was found to be an additional receptor for cysLTs. GPR17 can also be activated by uracil nucleotides [12]. There is a 38% sequence similarity between CysLT_1_R and CysLT_2_R and they are both able to induce calcium release upon activation [13], [14]. However, they do not appear to be functionally interchangeable and they might even antagonize each other's actions. For example, CysLT_2_R has been found to inhibit CysLT_1_R-induced proliferation in mast cells [15]. Colon tumors generally have an increased expression of CysLT_1_R [16] and a decreased expression of CysLT_2_R [17]. It has also been observed that colon cancer patients with high expression levels of CysLT_1_R have poor prognosis. Similar results are also seen in patients with breast cancer [18]. CysLT_2_R has, on the other hand, been implicated in the differentiation of Caco-2 cells [17] and is involved in vascular permeability [19]. This indicates that CysLT_2_R has a more protective role in cancer development while CysLT_1_R favors tumor progression.

Interferons (IFNs) are part of our defense system against viruses, bacteria, parasites, and malignant cells [20]. Both type I (for example IFN-α and IFN-β), and type II (IFN-γ) IFNs are produced during the early innate immune response. However, while IFN-γ is secreted mainly by natural killer cells and T-cells, the type I IFNs are produced by most cell types following virus infection or toll-like receptor activation [21]. IFN activation results in the increased activity of the transcription factor interferon regulatory factor 7 (IRF-7), which is involved in a positive feedback loop to augment a larger induction of IFN-α and IFN-β genes [22]. Even though both types of IFNs have been implicated to have anti-carcinogenic effects, the type I IFNs exhibit stronger anti-proliferative and anti-angiogenic effects and have been proposed to have apoptotic effects on cancer cells [23], [24]. In a mouse model, the type I IFNs have also been implicated to have anti-inflammatory functions in colitis [25]. In addition, they have demonstrated some effectiveness in the treatment of IBD patients [26]. We located a predicted IRF-7 binding site in the putative promoter region of the *CysLT_2_R*, which suggests that the *CysLT_2_R* promoter might be regulated by the IFN-α. This is interesting given that the anti-proliferative and anti-tumorigenic effects attributed to the IFN-α are consistent with the observed characteristics of the CysLT_2_R function [17]. IFN-γ has previously been reported to increase the expression of CysLT_2_R mRNA and protein in eosinophils [27]. In addition it enhances the cysLT-induced inflammatory response of primary endothelial cells [28].

Epidermal growth factor (EGF) signaling via the EGFR is a known inducer of cell proliferation and tumor cell invasion [29], [30]. Examining the putative promoter region for the CysLT_2_R, we located four conserved E-box elements (consensus sequence CANNTG). EGF signaling has been seen to induce transcriptional repression through E-boxes [31].

In this study, we found novel regulation factors for the *CysLT_2_R*. The anti-tumorigenic cytokine IFN-α is able to increase the transcription of the *CysLT_2_R* while EGF, a known inducer of cell proliferation and migration, suppresses the *CysLT_2_R* expression. This is consistent with the proposed anti-tumorigenic role for CysLT_2_R in colon cancer.

## Materials and Methods

### Chemicals

The rabbit polyclonal anti-human CysLT_2_R antibody (diluted 1∶1000) was purchased from Innovagen (Lund, Sweden). The ligand LTC_4_ and Montelukast were from Cayman Chemicals Co. (Ann Arbor, MI). The mouse anti-actin (diluted 1∶2000) and goat anti-IFNα/βR1 antibodies (diluted 1∶100, immunohistochemistry) were purchased from Sigma Chemicals Co. (St. Louis). The IFNαR1 antibody (diluted 1∶1000) was from Novus Biologicals (Litteleton, CO). The EGFR (diluted 1∶10, immunohistochemistry) was purchased from Zymed Laboratories Inc. (San Francisco, CA). EGFR (diluted 1∶800, Western blot) while the pEGFR (diluted 1∶1000) antibodies were from BD Transduction Laboratories (Erembodegem, Belgium). The total Snal 1 antibody (diluted 1∶500) was from Proteintech Group Inc. (Chicago, IL) and the pSnail antibody (diluted 1∶500) was from AbCam (Cambridge, UK). The secondary peroxidase linked goat anti-rabbit and anti-mouse antibodies (diluted 1∶5000) were from Dako (Glostrup, Denmark). Methyl-^3^H-thymidine, the enhanced chemiluminescence (ECL) reagents and the Western blot detection reagents as well as the hyperfilm were from Amersham International (Buckinghamshire, UK). TaqMan primers and master mix for real-time PCR were purchased from Applied Biosystems (Cambridge, UK). The RNeasy Plus Mini kit was from Qiagen GmbH (Hilden, Germany). Unless otherwise stated, all other reagents were of analytical grade and purchased from Sigma Chemicals Co. (St. Louis, MO) or from ICN (Temecula, CA).

### Cell Culture

The non-transformed human intestinal epithelial cell line (Int 407) [32], which exhibits typical epithelial morphology and growth, was cultured as a monolayer for approximately five days in Eagle's basal medium supplemented with 15% newborn calf serum. The two colon cancer cell lines, Caco-2 (DSMZ No: ACC 169) and SW 480 (DSMZ No: ACC 313), were respectively grown in Dulbecco's modified Eagle medium and RPMI 1640 supplemented with 10% and 20% fetal bovine serum respectively. All media was supplemented with 2 mM L-glutamine, 55 IU/mL penicillin and 55 µg/mL streptomycin. The cell lines were cultured at 37°C in a humidified atmosphere with 5% CO_2_. The cells were regularly tested to ensure the absence of mycoplasma contamination.

### Cell Lysates

Cells were washed, scraped with ice-cold 1×PBS, and centrifuged for 5 min at 1000 g. The cell pellets were lysed with lysis buffer [33] for 30 min on ice, homogenized 10 times with a 20G needle, and centrifuged for 5 min at 500 g.

### Gel Electrophoresis and Immunoblotting

To ensure equal loading, all samples were evaluated and compensated for protein concentration using the Coomassie blue protein assay. Proteins were denatured by boiling in sample buffer for 5 min [34]. The samples were subjected to electrophoresis on 8% or 10% polyacrylamide gels in the presence of 10% SDS. The immunoblotting and developing were performed as described in Nielsen *et al.* [34].

### Real-Time PCR Analysis

Cells were washed in PBS and immediately frozen at −80°C. Thereafter, they were scraped in lysis buffer provided in the kit and homogenized 10 times with a 20G needle. RNA was purified on RNeasy MinElute Spin Columns and dissolved in RNase free H_2_O. cDNA synthesis was performed using RevertAid H Minus M-MuLV reverse transcriptase (Fermentas Life Sciences). The mRNA expression levels of CysLT_2_R, CysLT_1_R, mucin-2 and the endogenous housekeeping gene, hypoxanthine phosphoribosyltransferase 1 (HPRT-1), were quantified using real-time PCR analysis (TaqMan Chemistry). cDNA was mixed with 0.9 µM TaqMan primers and master mix and amplified at 60°C in an Mx3005P thermocycler (Stratagene). The following primers were used: CysLT_2_R (Hs00252658_s1), CysLT_1_R (Hs00929113_m1), mucin-2 (Hs00159374_m1), and HPRT-1 (Hs99999909_m1). The samples were analyzed and normalized against HPRT-1 using the MxPro software (Stratagene).

### Plasmid Constructs

Human *CysLT_2_R* promoter fragments were subcloned into a pGL3-enhancer vector (Promega) containing a luciferase reporter gene. To produce construct I, one thousand base pairs upstream of the transcription start site of the *CysLT_2_R* gene were amplified from genomic DNA extracted from the human Int 407 cell line by nested PCR. The following primers were used in the process: forward 5′-ACATCAGGCAGCATTAATGT-3′and reverse 5′-GGACATAAATTTTCTCTCCAT-3′, followed. This was followed by a PCR that use the following primers; forward 5′-TTATGAGCTCGTTTCAAAACATTAAATG TAAC-3′ and reverse 5′-TTCTAAGCTTGCTGGGTTAAAAAGAAAC-3′. All PCR products were gel purified using the QIAquick gel extraction kit (Qiagen). They were digested with restriction enzymes and ligated into the pGL3-enhancer reporter vector using the Quick ligation kit (New England BioLabs). The plasmids were transformed into SoloPack Gold super competent cells (Stratagene). To produce the clone with the deleted IRF-7 binding site (construct II), the site directed mutagenesis with the following primers were used: forward 5′-ATGGCTATTCTACATTCAAAAATTATGAAATGTAATGCAGCATGT-3′ and reverse 5′-ACATGCTGCATTACATTTCATAATTTTTGAATGTAGAATAGCCAT-3′. The Quickchange XL kit (Stratagene) was used according to standard protocol. Three deletion constructs were produced by digesting construct I with restriction enzymes. Construct III was digested with *SacI* and *SpeI* to produce a construct from −1 to −412. Construct IV was digested with *HindIII* and *SpeI* to remove the downstream part of the promoter, thereby creating a construct between sites −1012 and −413 of the CysLT_2_R promoter. Blunt end digestion was performed using T4 DNA polymerase (Promega) and the vector was ligated back together. The promoter construct V was digested with *SacI* and *SmiI* to give a construct from −1 to −187. All plasmids were extracted using Endofree plasmid maxi kit (Qiagen) and were sequenced accordingly.

### Transient Transfections and Measurement of Luciferase Activity

Transient transfections of Int 407 cells were carried out using Lipofectamine LTX and Plus Reagent (Invitrogen) in serum free medium according to the manufacturer's instructions. The final DNA concentration used for transfections was 1 µg/ml for all plasmids except for the luciferase transfection control vector *Renilla* (0.2 µg/ml). The transfections were carried out for 4–6 h in 37°C, after which the medium was changed to 15% serum containing the medium. Forty-eight hours after transfection, the cells were stimulated with IFN-α (500–2000 U/ml) or EGF (100 ng/ml) in serum free medium for 24 h. The cells were lysed with passive lysis buffer (Promega) and stored in −80°C. Thawed lysates were centrifuged for 5 min at 1000 g and analyzed using the Dual Luciferase Reporter Assay System from Promega, following the manufacturer's instructions.

### Electromobility Shift Assay (EMSA)

Nuclear extracts were prepared from Int 407 cell treated with IFN-α 1000 U/ml or EGF 100 ng/ml for 24 h using the Nuclear Extraction Kit (Chemicon International). The process was performed according to the manufacturers' instructions. In additional, 10 mM 1-Naphthyl phosphate monosodium salt monohydrate, a broad phosphatase inhibitor, (Sigma Aldrich) was added into both cytoplasmic and nuclear lysis buffer before use. Biotin 3′ end-labeled single-stranded oligonucleotide corresponding to the IRF-7 binding region of *CysLT_2_R* putative promoter (sense: 5′-AAT CAG GAA ATT TAA ATT TAT TAT-3′ and antisense: 5′-ATA ATA AAT TTA AAT TTC CTG ATT-3′) or E-box binding site (sense: 5′-TTC TTT CAG CAT TTG AGA AAT GTG-3′ and antisense 5′-CAC ATT TCT CAA ATG CTG AAA GAA-3′) were purchased from TAG Copenhagen A/S. DNA probes were then annealed in buffer containing 10 mM Tris, 1 mM EDTA, and 50 mM NaCl (pH 8.0) by incubating the oligonucleotides at 95°C for 5 min then gradually reducing the heat until the DNA reached room temperature. Binding reactions for EMSA were prepared using the LightShift Chemiluminescent EMSA kit (Pierce Thermo Scientific). The binding buffer for IRF-7 contained 10 mM Tris-HCl (pH 7.5), 1 mM EDTA, 50 mM NaCl, 2 mM dithiothreitol, 5% glycerol, 0.5% NP-40, and 10 µg/ml BSA. To reduce nonspecific binding, 62.5 µg of poly(dI-dC) was added per ml. Each reaction contains 20 µg nuclear extract. After 20 min of incubation with the probe at room temperature, extracts were loaded on a 5% polyacrylamide gel. After 1 h at 100 V, the gel was transferred onto a positive charged Nylon Membrane, (Thermo scientific) and detected according to kit instruction. The binding buffer for E-box contained 20 mM HEPES, pH 7.6, 150 mM KCl, 3 mM MgCl_2_, 10% glycerol, 0.2 mM ZnSO_4_, 0.3 mg/ml BSA) and 1 µg/20 µL of poly(dI∶dC). The binding reaction was performed on ice for 30 min before being loaded onto the gel. To demonstrate the specificity of protein-DNA complex formation, a 1,000-fold molar excess of unlabeled oligonucleotides or IRF-7 (G-8) antibody (Santa Cruz) or SNAI 1 (E-18) antibody (Santa Cruz) was added to the binding reaction mixtures before the probe.

### Alkaline Phosphatase Activity

The assay was performed as previously described [35]. Briefly, alkaline phosphatase activity was measured using disodium p-nitrophenyl phosphate as substrate. Caco-2 cells were seeded in Petri dishes and incubated for 24 h at 37°C. Five hundred to 2000 U/ml of IFN-α and/or 40 nM of LTC_4_ was added to the cells and subsequently incubated for 72 h at 37°C. New LTC_4_ was added every 24 h. Sodium butyrate (2 mM) was used as a positive control (data not shown). The alkaline phosphatase activity was estimated by measuring the level of p-nitrophenol at 405 nm.

### Thymidine Incorporation Assay

Caco-2 cells were grown in flat-bottomed 96 well plates for five days. The medium was changed to serum-free 2 h prior to stimulation. Cells were stimulated with LTC_4_ (40 nM), IFN-α (1000 U/ml) or EGF (100 ng/ml) for 48 h. DNA synthesis was assessed by adding 0.5 µCi of [^3^H] thymidine during the final 18 h of stimulation. The wells were washed once with PBS and incubated with 0.05% trypsin-EDTA solution/well for 5 min at 37°C. Cells were harvested and collected on a filter paper in a Perkin Elmer harvester. The filter paper was dried and [^3^H] thymidine incorporation was measured in a 1450 Microbeta Trilux liquid scintillation counter (Wallac).

### Cell Migration Assay

Cell migration was analyzed in a modified Boyden chamber, which consisted of two chambers separated by a polycarbonate PVDF membrane (pore size of 8 µm) that was covered with a collagen I gel (3 mg/ml). Int 407 cells were grown for 18 h in the presence or absence of LTC_4_ (40 nM) and/or EGF (100 ng/ml) in medium containing 1.5% serum. Fifteen percent serum was used as a positive control. Cells were added to the upper well and allowed to migrate into the chamber for 18 h at 37°C. The migrated cells were fixed in 4% paraformaldehyde and the membranes were stained with a 1% crystal violet/10% in a methanol solution. The membranes were washed in PBS and the remaining dye was removed with 10% SDS. The absorbance was measured at 590 nm.

### Patient Samples

Archival formalin fixed and paraffin embedded colon cancer and control colon specimens of colorectal cancer patients collected in 1990 were obtained from the biobanks of Malmö University Hospital. Tissues from 78 patients with varying grades and stages of the disease were included. Grading of the tumors was performed using Dukes' classification [36]. The matched control samples of normal colon tissue in this investigation were taken from the borders of the surgical specimens.

### Ethics Statement

This study was performed after ethical permission from the Regional Ethical Review Board, Lund University # 367/2005. Archival tissue specimens from 78 colon cancer patients that were operated between 1990 and 1991 were used in the present study. Since the samples were old and taken from different parts of the region, it was not possible to obtain written consent from each patient. Detailed information describing the study and tissue microarray (TMA) construction was published in 2006 in a daily newspaper and patients were offered to contact us by mail or by phone if they had any objections. None of the 78 patients objected. This procedure was performed following strict guidelines from the Regional Ethical Review Board in Lund who approved the procedure.

### Tissue Arrays and Immunohistochemistry

For histological assessment, the archival paraffin-embedded colorectal cancer and normal mucosa specimens were used and prepared as previously described [16]. The array was stained with anti-IFNα/βR1, EGFR and CysLT_2_R antibodies. Secondary peroxidase antibodies used were from Dako. After immunostaining, all slides were counterstained with Mayer's hematoxylin.

### Statistical Analyses

Statistical significance was determined as P<0.05 by two-tailed Student's t test or one-way ANOVA. Pearson's correlation test was used when comparing different sets of immunohistochemistry staining. SPSS software 16,0 (SPSS, Inc.) was used for the patient material analysis.

## Results

### An IRF-7 Binding Site and Four E-boxes Have Been Found to be Present in the *CysLT_2_R* Promoter Region

**Figure 1:**
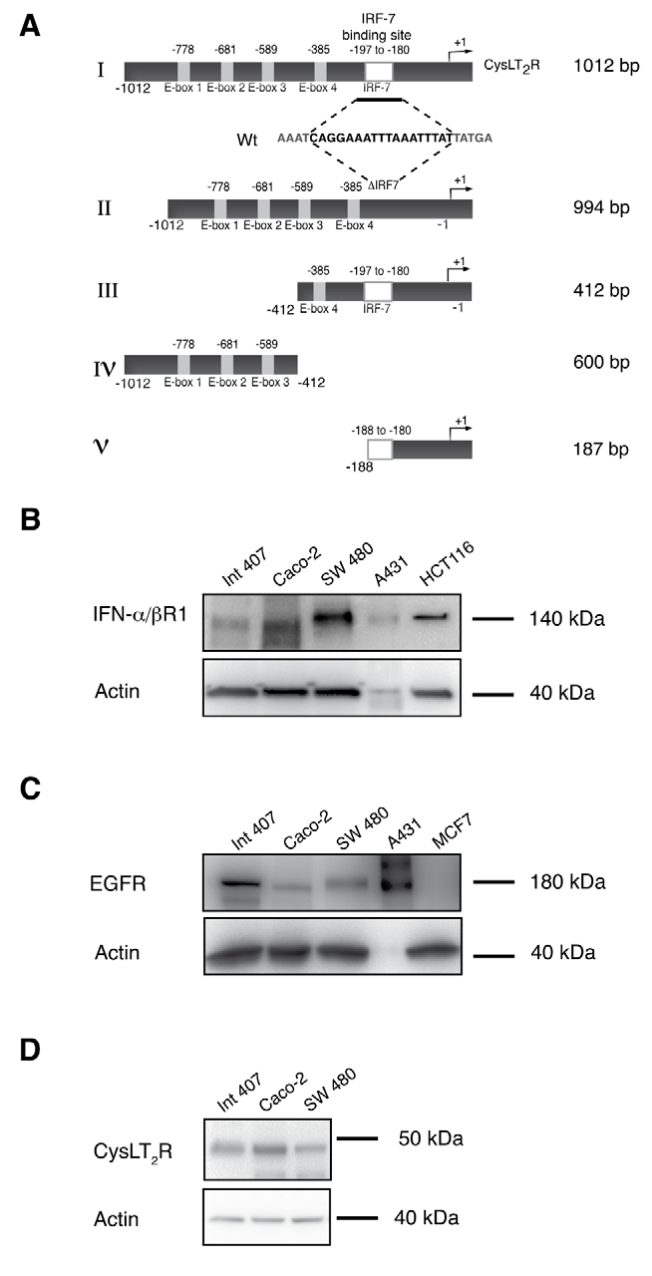
There is an IRF-7 binding site and four E-boxes present in the *CysLT_2_R* putative promoter region. (**A**) Schematic visualization of the *CysLT_2_R* promoter. Construct I contains the wildtype promoter region (1012 bp) and constructs II–V are various deletion constructs. Representative Western blots from three different experiments with the intestinal epithelial cell line Int 407 and the colon cancer cell lines Caco-2 and SW 480 were analyzed as follows. (**B**) The blot shows the levels of IFNαR1, and A431 cells were used as a negative control while HCT116 cells were used as a positive control. (**C**) The blot shows the levels of EGFR protein expression, A431 cells were used as a positive control and MCF7 cells were used as a negative control, and (**D**) the blot shows the levels of CysLT2R protein expression.

To increase the understanding of the role of cysteinyl leukotrienes in inflammation-induced colorectal carcinogenesis, we examined possible ways by which the expression of CysLT_2_R can be regulated, with one of the receptors generating the effects of leukotrienes. The promoter region of CysLT_2_R contains several interesting binding sites for known transcription factors and transcriptional repressors. Previous reports from our laboratory have shown that CysLT_2_R is down regulated in colon cancer [17], [37] and that it is involved in colon cancer cell differentiation [17] as well as in decreased cell migration in breast cancer cells [18]. Therefore, it was of particular interest that a putative binding element for IRF-7 and four E-box elements were present in the promoter region of the CysLT_2_R. Five constructs of the CysLT_2_R promoter region were created and ligated into a pGL3-enhancer reporter vector to evaluate the importance of IRF-7 and the E-boxes for activation/transcription of *CysLT_2_R* (Fig. 1A). IFN-α can be used to activate the transcription factor IRF-7. However, the presence of the IFN-α receptor IFN-αR1 is needed to do this. The presence of the IFN-αR1 is confirmed in the intestinal cell lines intended for this study (Fig. 1B). Likewise, the expression of the EGFR (Fig. 1C) and the CysLT_2_R (Fig. 1D) was verified.

### *CysLT_2_R* Promoter Activity is Activated by the IFN-α via an IRF-7 Binding Site and It is Repressed by the EGF Through the E-boxes Present in the Putative Promoter Region

**Figure 2:**
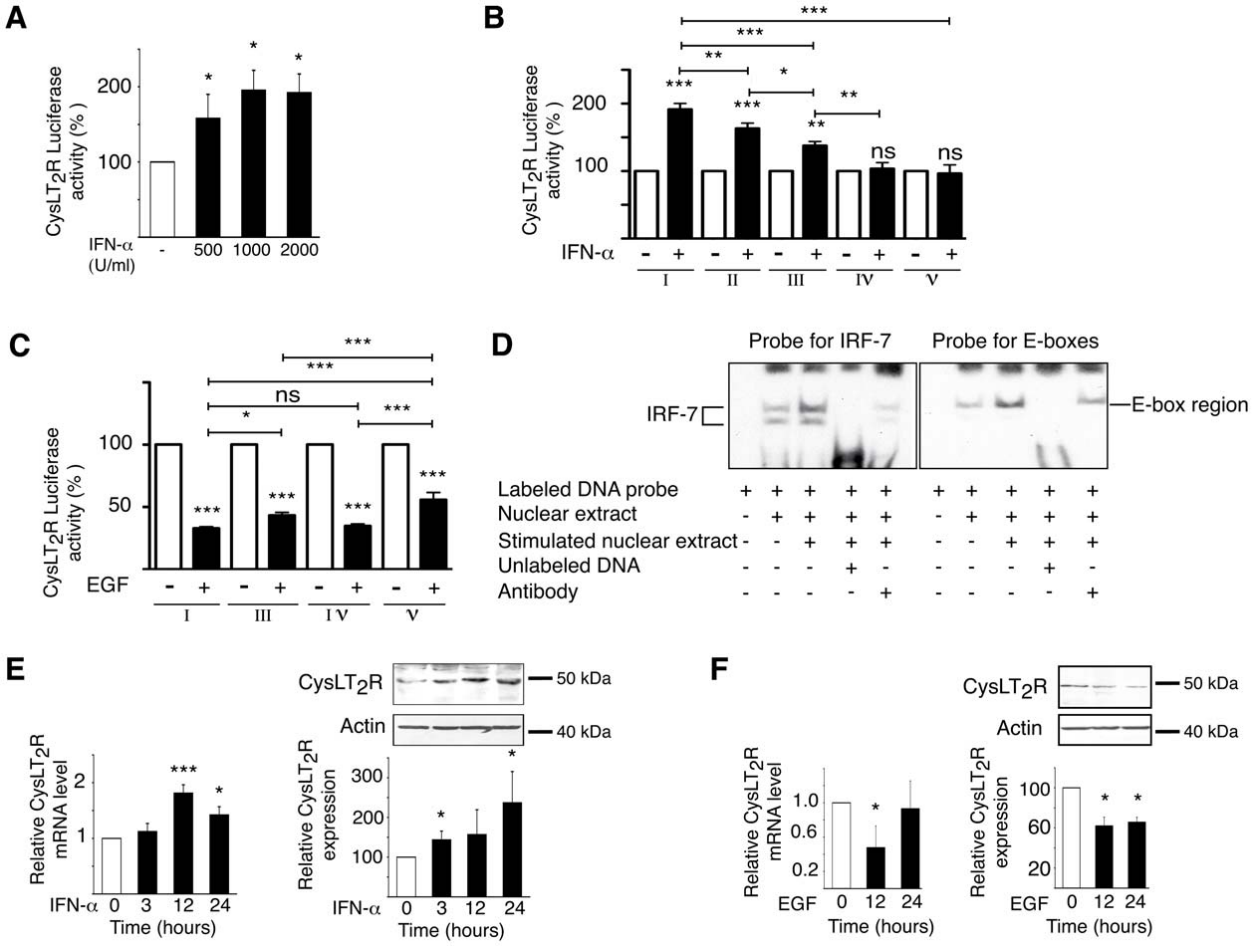
IFN-α induces *CysLT_2_R* luciferase reporter activity whereas EGF suppresses it. (**A**) *CysLT_2_R* luciferase activity following transient transfection with pGL-3-CysLT_2_R construct in Int 407 cells, with or without treatment with 500–2000 U/ml IFN-α for 24 h. (**B**) Int 407 cells transfected with wildtype *CysLT_2_R* promoter or *CysLT_2_R* promoter deletion constructs. Cells were grown in the presence of IFN-α (1000 U/ml) or in the presence of EGF (100 ng/ml) for 24 h (**C**). In brief, followed by stimulation with IFN-α or EGF for 24 h and measurement of luciferase activity (relative luciferase units). Results shown are the mean ± SD of at least three independent experiments performed in triplicate. (**D**) Analysis of IRF-7 and E-box DNA binding activities by EMSA on *CysLT_2_R* promoter region in Int 407 cells. Modulation of IRF-7 DNA binding in cells stimulated with IFN-α (1000 U/ml). For competition assays, a 1000-fold molar excess of unlabeled oligonucleotides (Lane 4) or 10 µg anti-IRF-7 antibody (Lane 5) was added before addition of probe to the binding reactions, (**left panel**). Effect on E-box DNA binding in cells stimulated with 100 ng/ml EGF for 24 h. A 1000-fold molar excess of unlabeled oligonucleotides (Lane 4), or 10 µg anti-Snail antibody was added before addition of probe to the binding reactions (**right panel, lane 5**). (**E**) Real-time PCR quantification of mRNA expression of CysLT_2_R and immunoblot analysis of CysLT_2_R protein following treatment with or without 1000 U/ml IFN-α. (**F**) Real-time PCR quantification of mRNA expression of CysLT_2_R and immunoblot analysis of CysLT_2_R protein with or without treatment with EGF (100 ng/ml). The results are shown as means ± SD of at between three to teen separate experiments; *, P<0.05; **, P<0.01; ***, P<0.001 for one-way ANOVA or two tailed Student's t-test.

We have seen that CysLT_2_R expressions were altered in patient's tumor material, which might be due to a changed “microenvironment around the tumor”. Therefore, we decided to search for a Th1 regulatory cytokine motif in the promoter region of the *CysLT_2_R*. The presence of a putative IRF-7 binding site in the CysLT_2_R promoter region indicated that the Th1 pro-inflammatory cytokine IFN-α ought to induce *CysLT_2_R* promoter activity and transcription of the *CysLT_2_R* encoding gene. A dilution series with IFN-α showed that 1000 U/ml IFN-α generated an optimal activation of *CysLT_2_R* (Fig. 2A). To elucidate whether IFN-α induces the *CysLT_2_R* through IRF-7 binding to its putative binding site in the promoter region, the promoter activity for deletion mutant constructs I-V were tested (Fig. 2B). IFN-α induced a significant two-fold activation of the *CysLT_2_R* in the non-transformed cell line Int 407 (construct I). This activation was partly reduced in the ΔIRF-7 mutant (construct II). IFN-α was unable to induce the *CysLT_2_R* promoter activity in constructs IV and V, which both lacked full-length IRF-7 response elements. However, a significant reduction in activation compared to wild-type construct I was also observed for construct III, which contains an intact IRF-7 response element but lacks the part upstream of bp −412. Taken together, this indicates that the IFN-α-induced activation of the *CysLT_2_R* requires the IRF-7 binding site for complete activation, but also needs additional unidentified binding elements located up-stream of the IRF-7 site for full activation.

Further examination of the *CysLT_2_R* promoter sequence revealed the presence of four conserved E-box elements. EGF signaling can induce repressor elements that bind to mentioned sequences. Four of the *CysLT_2_R* luciferase reporter constructs were used to investigate the effects of EGF on the *CysLT_2_R*. We found that EGF suppressed *CysLT_2_R* luciferase reporter activity (Fig. 2C). The importance of the E-boxes was investigated by testing the ability of the EGF to suppress *CysLT_2_R* promoter activity in three deletion constructs, (construct III, with only E-box 4 present, construct IV containing three E-boxes, and construct V without any E-boxes). The EGF-induced reduction of *CysLT_2_R* promoter activity significantly declined with a decreasing number of E-boxes present in the putative promoter region. There was a statistically significant difference between the EGF-induced reductions of construct IV and construct V. However, the reduction is probably mediated both through an E-box-dependent as well as an E-box-independent pathway in the intestinal cells.

Analysis of IRF-7 and E-box DNA binding activities on the *CysLT_2_R* putative promoter region in Int 407 cells were performed to compliment the luciferase experiments (Fig. 2D). The EMSA results showed that IRF-7 containing complexes were detected in both untreated and IFN-α treated cells. We found an increased association upon the stimulation of the complexes. The addition of cold probe and anti-IRF-7 antibody competed with IRF-7 in binding the IRF-7 binding site on the promoter region. The addition of cold probe could completely inhibit probe-protein complex formation. Likewise, Snail-containing complexes were detected in both untreated and EGF treated cells. We found increased association upon stimulation of the complex. The addition of cold probe could completely inhibit the formation of probe-protein complex. Moreover, additional anti-Snail antibody reduced the probe-protein complex formation.

Next, we wanted to see that the regulatory effects for IFN-α and EGF observed in connection with *CysLT_2_R* luciferase reporter activity also affected CysLT_2_R mRNA and protein expression. Consistent with the results from the gene activation assay, the IFN-α significantly up-regulated both the CysLT_2_R mRNA (peaking at 12 h) and the protein (maximum increase at 24 h) (Fig. 2E). The EGF induced a significant suppression of both the CysLT_2_R mRNA and the protein expression (Fig. 2F).

### EGFR and Snail Activation is Involved in EGF Repressing *CysLT_2_R*

**Figure 3:**
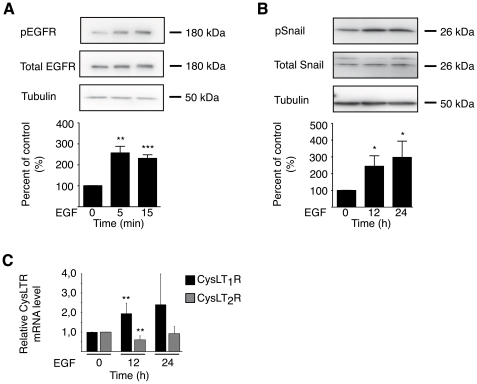
EGF induced activation of EGFR and snail in Int 407 cells. (**A**) Western blot of Int 407 cells showing phosphorylation of EGFR after EGF (100 ng/ml) treatment for 5 or 15 min. (**B**) Western blot of Int 407 cells showing phosphorylation of snail after EGF treatment (100 ng/ml) for 12–24 h. (**C**) Real-time PCR quantification of mRNA expression of CysLT_1_R and CysLT_2_R with and without EGF (100 ng/ml) treatment. The results are shown as means ± SD of at least three separate experiments; *, P<0.05; **, P<0.01 and ***, P<0.001.

Stimulation with EGF leads to the activation of the EGFR (Fig. 3A) and to the activation of Snail (Fig. 3B), a transcriptional repressor known to bind to E-box elements. It is likely that the EGF-induced suppression of the CysLT_2_R is generated by Snail that binds to the E-box elements present in the promoter region of the *CysLT_2_R*. Interestingly, an induction of CysLT_1_R mRNA is observed after EGF treatment of intestinal cells at the same time as CysLT_2_R mRNA is suppressed (Fig. 3C), indicating, as earlier suggested, opposing effects of the CysLTRs [37].

### IFN-α Can Induce Differentiation of Caco-2 Cells

**Figure 4:**
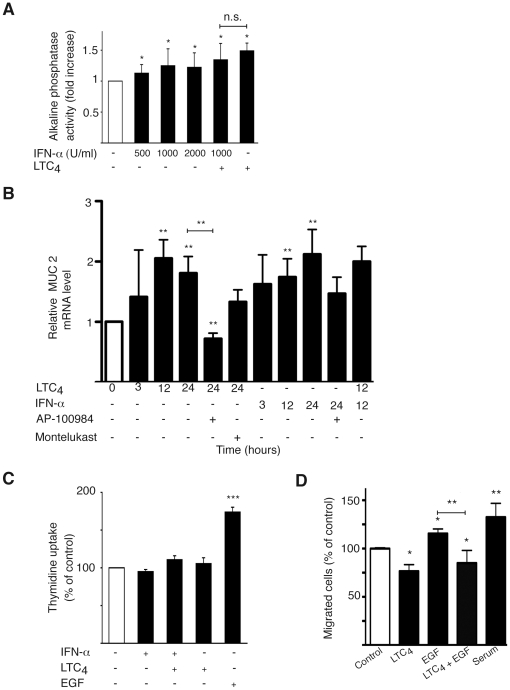
CysLT_2_R signaling mediates anti-tumorigenic effects. (**A**) An alkaline phosphatase activity assay was used to determine the differentiation of Caco-2 cells. Cells were treated with IFN-α (500–2000 U/ml) and/or LTC_4_ (40 nM) for 72 h. The alkaline phosphatase activity was determined by measuring the absorbance at 405 nm due to formation of *p*-nitrophenol. (**B**) QPCR quantification of mRNA expression of MUC2 with or without treatment with LTC_4_ (40 nM), IFN-α (1000 U/ml), pretreatment for 30 min with CysLT_2_R inhibitor AP-100984 (1 µM) or CysLT_1_R inhibitor Montelukast (1 µM), in Caco-2 cells. (**C**) Caco-2 cells were incubated with LTC_4_ (40 nM) and/or IFN-α (1000 U/ml) for 48 h in medium containing 1.5% serum. To determine proliferation by thymidine uptake, [methyl-^3^H] thymidine (0.5 µCi/well) was added to the wells during stimulation. (**D**) Cell migration was analyzed with Int 407 cells grown in the presence or absence of EGF (100 ng/ml) and/or LTC4 (40 nM). The cells were allowed to invade a collagen gel on top of a Boyden chamber for 18 hrs. The results are shown as means ± SD of at least three different experiments; *, P<0.05; **, P<0.01; ***, P<0.001.

Sodium butyrate is a known inducer of differentiation in Caco-2 cells [38], which is frequently used as a model system for differentiation in cell culture [39]. The activation of different luminal brush border proteins, such as alkaline phosphatase, can be used as a measurement for epithelial cell differentiation. Previously, we have shown that the CysLT_2_R ligand LTC_4_ is able to induce differentiation in Caco-2 cells [17]. IFN-α induced a small but significant increase in the activation of alkaline phosphatase, indicating increased differentiation of Caco-2 cells. IFN-α had, however, no additive effect to the alkaline phosphatase activity induced by LTC_4_ (Fig. 4A). To confirm the effect of alkaline phosphatase activity, an additional differentiation assay was used. Mucin-2 is a marker for intestinal cell differentiation [40]. Both LTC_4_ and IFN-α could significantly induce mucin-2 mRNA in Caco-2 cells (Fig. 4B). The CysLT_2_R antagonist AP-100984 could significantly block both LTC_4_ induction of mucin-2, which was not seen with the specific CysLT_1_R antagonist Montelukast. This strengthens the hypothesis that CysLT_2_R might have a protective role in colon cancer and in normal intestinal mucosa. We have previously shown that LTC_4_ does not induce proliferation in Caco-2 cells [17]. Here, we show that IFN-α does not induce a proliferative response either. EGF, on the other hand, is a known mitogen inducer [41] and was used here as a positive control (Fig. 4C). This corresponds well to the previous reported roles of the CysLTRs.

We next investigated the effect of CysLT_2_R signaling on Int 407 cell migration. We found that LTC_4_-induced activation of CysLT_2_R reduced Int 407 cell migration (Fig. 4D). Furthermore, we also found that EGF-induced Int 407 cell migration could be suppressed by CysLT_2_R activation (Fig. 4D).

### EGFR Inversely Correlates with CysLT_2_R in Colorectal Cancer

**Figure 5:**
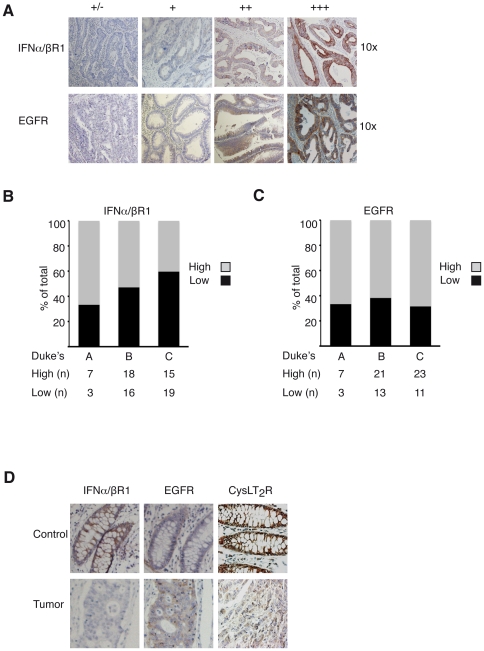
Representative IFNα/βR1 and EGFR staining in normal human colon tissue and colorectal adenocarcinomas. (**A**) Top row, shows the degree of IFNα/βR1 staining of carcinomas. Bottom row shows the degree of EGFR staining of carcinomas (+/− to +++, 10× objective). (**B**) Distribution of high (++, +++) and low (+/−, +) IFNα/βR1 staining intensities of tumors in Duke's A, B and C, and (**C**) of EGFR. Samples are assessed according to total IFNα/βR1 and EGFR staining. Statistics are based on tumors from 78 patients that were included in the array. (**D**) Representative pairs of control and tumor immunostaining from a Duke's C patient stained with IFNα/βR1, EGFR and CysLT_2_R.

Next, we wanted to clarify if the levels of IFNα/βR1 and EGFR correlated with the CysLT_2_R pattern in a colon cancer patient array. The TMA contains material from 78 colon cancer patients and has previously been thoroughly described [16]. Representative stainings from the array for IFNα/βR1 and EGFR are shown in Fig. 5A. We have previously shown that CysLT_2_R is down-regulated in colon tumor tissue in patients from this array and that more aggressive tumors expressed less of the CysLT_2_R [17]. We observed that patients with more aggressive tumors generally had less expression of IFNα/βR1 while patients with less aggressive tumors generally exhibited a higher expression of IFNα/βR1. A more distinct trend was visualized when the patients were divided into subgroups with high (++, +++) or low (+/−, +) IFNα/βR1 staining. Duke's A patients had a higher percentage of increased IFNα/βR1 staining than Duke's B and Duke's C patients (Fig. 5B). The expression of the EGFR was generally up-regulated in colon cancer patients in this study, which is coherent with earlier published studies [42] (Fig. 5C). Representative pictures from a Duke's C colon cancer patient are shown in Fig. 5D. They exhibit high expression of CysLT_2_R and IFNα/βR1 in control tissue and decreased expression in the tumor. Conversely, EGFR expression was enhanced in the tumor material. A statistical significant negative correlation could be observed in the expression levels between CysLT_2_R and EGFR in this tissue array. Although IFNα/βR1 and CysLT_2_R had similar expression patterns in relation to cancer staging, no significant correlation between the expression levels of these two receptors could be found (Table 1).

**Table 1:** CysLT_2_R immunoreactivity vs. IFNα/βR1 and EGFR.

		CysLT_2_R						
Relative Staining	Frequency						Spearman's rank	p
	(% of total)	+/−	+	++	+++	n	Correlation	
**Frequency**		11	28	31	7	77		
**IFNa/bR1**								
**+/−**	2.8	0	1	1	0	2	0.062	**0.608**
**+**	40.8	7	7	13	2	29		
**++**	35.2	2	11	10	2	25		
**+++**	21.1	1	8	5	1	15		
**EGFR**								
**+/−**	1.4	0	1	0	0	1	−0.392	**0.001**
**+**	36.6	0	9	14	3	26		
**++**	36.6	3	12	9	2	26		
**+++**	25.4	7	5	6	0	18		

## Discussion

Several different ways of regulating CysLT_2_R have previously been reported. For example, LTD_4_ can up-regulate the mRNA expression of CysLT_2_R in monocytes [43] and epithelial cells [44] and the cytokines IL-4, IL-8 and IFN-γ [28], [45], [46] can up-regulate the mRNA expression of CysLT_2_R in several different cell types. Since a binding site for IRF-7 was present in the putative promoter region of the *CysLT_2_R*, we hypothesized that the IFN-α would be able to induce CysLT_2_R expression via transcription factor IRF-7. The ability of the IFN-α to induce anti-proliferative and anti-tumor progression responses [24], [47] made it an interesting candidate to study in relation to CysLT_2_R. IFNs have been reported to mediate their anti-tumor effects by altering immune responses, such as the suppression of cytokine IL-1 [48] and inducing TRAIL [49], and by inducing anti-angiogenic responses by inhibiting VEGF [50]. We show that the IFN-α induced a two-fold activation of the wild-type *CysLT_2_R* promoter and increased expression in the CysLT_2_R mRNA and protein. How the IFN-α mediates its effects, however, is not completely elucidated. The IRF-7 binding site is important and required for the optimal induction of the CysLT_2_R promoter by the IFN-α, but it is not the only crucial activation site. Additional transcription factors are probably engaged as well. We discovered that the promoter region of the *CysLT_2_R* contains four conserved E-box elements. Interestingly, the mitogenic EGF suppressed both CysLT_2_R mRNA and protein expression as well as the promoter activity, strengthening the hypothesis that the CysLT_2_R is a potential tumor suppressor. To validate the importance of the E-boxes, *CysLT_2_R* promoter deletion constructs were used. EGF stimulation significantly decreased the *CysLT_2_R* promoter activity in all promoter constructs, but to a significantly reduced capacity in the deletion constructs. These results indicate that the E-box elements are important but are not the only mechanisms that allow the EGF to suppress the transcription of the *CysLT_2_R*. We cannot rule out that other transcription factors can also be activated by the EGF to reduce the *CysLT_2_R* luciferase reporter activity of construct V that lack the E-boxes. Mann *et al.* report that EGF, through the EGFR, can repress the proposed tumor suppressor prostaglandin dehydrogenase (PGDH), allowing PGE_2_ to accumulate and activate the pathway repeatedly in a positive feedback loop. They show that EGFR signaling induces Snail, which binds to the conserved E-box elements in the *PGDH* promoter and represses transcription [31]. It is likely that the repression of the *CysLT_2_R* in the presence of EGF is a result of EGF signaling via the EGFR, thereby activating the Snail, which then functions as a transcriptional repressor when bound to the E-boxes present in the *CysLT_2_R* promoter region. Earlier, we have shown that LTC_4_ is able to induce the differentiation of Caco-2 cells [17]. In this study, we show that IFN-α is also able to induce differentiation in colon cancer cells. This was demonstrated by the induction of mucin-2 after IFN-α stimulation. Mucins are highly glycosylated proteins that build up the mucus layer protecting the underlying epithelial cells in the intestine. The colon epithelium mainly secretes mucin-2 [51], which is known to play a protective role against colorectal diseases, such as colon cancer [52] and colitis [53]. Mucin-2-deficient mice frequently develop adenomas, which progress into invasive adenocarcinomas, demonstrating that mucin-2 is involved in colon cancer suppression [52]. Interestingly, we demonstrate that both LTC_4_ and IFN-α significantly induced the mRNA expression of mucin-2. Taken together, this implicates that both LTC_4_ and IFN-α are involved in the stimulation of mucus synthesis and are important in protecting against mucosal damage in the colon. We have previously shown that endogenous signaling by overexpressed CysLT_2_R is enough to suppress cell migration in breast cancer cells [18]. Here, we show that LTC_4_ signaling mediates a suppression of cell migration and reduces the cell migration induced by EGF signaling. The ability of CysLT_2_R signaling to suppress cell migration is probably one of the reasons why high CysLT_2_R expression is connected to a better prognosis for colorectal cancer patients. It is possible that the balance between positive and negative gene regulators, such as repressors and transcription factors and their co-factors, are disrupted in the tumor microenvironment. This could explain the manner by which the down-regulation of CysLT_2_R plays a role in colon cancer progression.

We observed a decrease of IFNα/βR1 in tumors in colon cancer patients. Considering the protective role that IFNs play in the innate immune system (i.e. participating in the cell defense against infections and tumor cells), we speculate that tumor cells have an impaired innate immune response. There are epidemiological associations between pathogen invasion leading to chronic inflammation and malignant transformations [2]. For example, virus infections have been connected to increased incidences of cancer. Infection with hepatitis B or C viruses can result in chronic hepatitis and liver cirrhosis, which are believed to be a major cause of hepatocellular carcinoma [2]. In addition, the IFN-α can achieve anti-tumorigenic effects by affecting tumor cell differentiation [47]. The observation that IFNα/βR1 had lower expression in more aggressive tumors agrees with an anti-tumorigenic role for IFN-α. Furthermore, the expression of IFNα/βR1 was reduced in tumor tissue compared to normal tissue from the surgical borders of the tumor in the patients, contributing to the notion of altered IFN signaling in tumor cells. The same clinical material displayed overexpression of EGFR in tumor tissue. The expression pattern for CysLT_2_R [17] is similar to the expression of anti-tumorigenic IFNα/βR1while the expression of CysLT_1_R is increased in colon cancer [16] similar to the expression of EGFR. Both CysLT_1_R and EGFR exhibit pro-tumorigenic effects by driving the processes of cell migration and proliferation [29], [30], [33], [54]. The nuclear localization of EGFR has been linked to more aggressive tumor types [29] and increased nuclear localization of CysLT_1_R has also been observed in colon cancer cells [34]. We show that EGF signaling induces CysLT_1_R mRNA while suppressing CysLT_2_R. Interestingly, EGFR and CysLT_2_R display a significant inverse correlation in their expression pattern among the colon cancer patients in the array. In conclusion, we have located one response element for IRF-7 and four E-boxes in the promoter region of *CysLT_2_R*. We demonstrate that the anti-tumorigenic IFN-α is able to induce *CysLT_2_R* promoter activity and expression while EGF, a known inducer of mitogenic effects, is able to suppress it. These data support the hypothesis that CysLT_2_R might have a protective role in colon cancer.
